# Role of Bacterial Vaginosis in Peripartum Infections

**DOI:** 10.1155/S106474499400061X

**Published:** 1994

**Authors:** Penny Clark, Traci Kurtzer, Patrick Duff

**Affiliations:** Division of Maternal Fetal Medicine Department of Obstetrics & Gynecology University of Florida, College of Medicine P.O. Box 100294 Gainesville FL 32610-0294 USA

## Abstract

*Objective:* The purpose of this prospective investigation was to determine if the presence of bacterial vaginosis (BV) at the time of delivery was associated with the development of maternal and neonatal infection.

*Methods:* Vaginal fluid was collected from 390 laboring patients. Smears of the vaginal secretions were gram stained, and slides were scored and interpreted as normal, intermediate, and BV based on Gram's stain criteria. Results of the Gram's stains were correlated with the clinical diagnoses of chorioamnionitis, endometritis, and neonatal sepsis.

*Results:* Eighty-eight percent of patients were term and 12% were preterm. The overall prevalence of BV was 30%. The frequency of BV was similar in both term and preterm women. BV was significantly more prevalent among nonwhites than whites (37% vs. 25%, *P* = 0.005). Maternal characteristics such as mean age, parity, status of the membranes, mean duration of labor, mean duration of ruptured membranes, mean length of fetal monitoring, mean number of vaginal examinations, and mode of delivery were similar in patients with BV, intermediate, and normal Gram's stains. Forty-seven (12%) women developed peripartum infection. The frequencies of chorioamnionitis or endometritis in women with BV or intermediate Gram's stains were 19/116 (16.4%) and 11/63 (17.5%), respectively. The frequency in each of the 2 groups was significantly increased compared with the rate in women with normal Gram's stains: 17/211 (8.1%), [*P* = 0.034, OR = 2.0 (95% CI, 1.07–3.73) for BV and *P* = 0.054, OR = 2.1 (95% CI, 1.12–3.94) for intermediate Gram's stain]. The incidence of suspected or confirmed neonatal infection was significantly higher in mothers with intermediate Gram's stains compared with mothers with normal Gram's stains (*P* = 0.02, OR = 2.18, 95% CI, 1.12–3.94), while no difference in incidence was observed between mothers with BV and normal Gram's stains (*P* > 0.05). The rate of neonatal infection directly correlated with maternal group B streptococcal colonization rather than with BV.

*Conclusions:* In this population, patients with BV and intermediate Gram's stains had an increased frequency of peripartum infection.


Infectious Diseases in Obstetrics and Gynecology 2:179–183 (1994)
© 1994 Wiley-Liss, Inc.

### Role of Bacterial Vaginosis in Peripartum Infections

**Penny Clark, Traci Kurtzer, and Patrick Duff**

*Division of Maternal Fetal Medicine, Department of Obstetrics & Gynecology, University of Florida College of Medicine, Gainesville, FL*

### Abstract

***Objective:*** **The purpose of this prospective investigation was to determine if the presence of bacterial vaginosis (BV) at the time of delivery was associated with the development of maternal and neonatal infection.**

***Methods:*** **Vaginal fluid was collected from 390 laboring patients. Smears of the vaginal secretions were gram stained, and slides were scored and interpreted as normal, intermediate, and BV based on Gram's stain criteria. Results of the Gram's stains were correlated with the clinical diagnoses of chorioamnionitis, endometritis, and neonatal sepsis.**

***Results:*** **Eighty-eight percent of patients were term and 12% were preterm. The overall prevalence of BV was 30%. The frequency of BV was similar in both term and preterm women. BV was significantly more prevalent among nonwhites than whites (37% vs. 25%, $P = 0.005$). Maternal characteristics such as mean age, parity, status of the membranes, mean duration of labor, mean duration of ruptured membranes, mean length of fetal monitoring, mean number of vaginal examinations, and mode of delivery were similar in patients with BV, intermediate, and normal Gram's stains. Forty-seven (12%) women developed peripartum infection. The frequencies of chorioamnionitis or endometritis in women with BV or intermediate Gram's stains were 19/116 (16.4%) and 11/63 (17.5%), respectively. The frequency in each of the 2 groups was significantly increased compared with the rate in women with normal Gram's stains: 17/211 (8.1%), [$P = 0.034$, OR = 2.0 (95% CI, 1.07–3.73) for BV and $P = 0.054$, OR = 2.1 (95% CI, 1.12–3.94) for intermediate Gram's stain]. The incidence of suspected or confirmed neonatal infection was significantly higher in mothers with intermediate Gram's stains compared with mothers with normal Gram's stains ($P = 0.02$, OR = 2.18, 95% CI, 1.12–3.94), while no difference in incidence was observed between mothers with BV and normal Gram's stains ($P > 0.05$). The rate of neonatal infection directly correlated with maternal group B streptococcal colonization rather than with BV.**

***Conclusions:*** **In this population, patients with BV and intermediate Gram's stains had an increased frequency of peripartum infection.** © 1994 Wiley-Liss, Inc.

Key words

Vaginal infections, chorioamnionitis, puerperal endometritis

Bacterial vaginosis (BV) is a common vaginal infection characterized by an alteration of the normal vaginal flora such that the naturally predominant species of *Lactobacillus* are replaced by *Gardnerella vaginalis*, *Bacteroides* sp., *Prevotella* sp., *Mobiluncus* sp., and *Peptococcus* sp.[1–3] BV has been associated with several adverse obstetric outcomes. Martius et al.[4] found an increased frequency of preterm premature rupture of membranes in women with BV. Gravett et al.[5] reported that the bacteria recovered from the amniotic fluid of women delivering preterm included many of the same organisms responsible for BV. Krohn and coworkers[3] observed high concentrations of *Prevotella bivia* and *Bacteroides fragilis* in the genital tracts of patients who delivered preterm. BV has

Address correspondence/reprint requests to Dr. Patrick Duff, Department of Obstetrics & Gynecology, P.O. Box 100294, University of Florida, Gainesville, FL 32610-0294.

Received May 2, 1994
Accepted August 9, 1994

Clinical Study also been implicated in the development of both intrapartum and postpartum infection. In a report by Watts et al.[6], women with BV were 6 times more likely to develop endometritis after cesarean delivery than women without BV. The association between BV and puerperal infection, however, has not been fully established. Accordingly, in a large prospective investigation, we attempted to determine if a direct correlation exists between BV and intrapartum and postpartum infection.

### SUBJECTS AND METHODS

Our investigation was conducted at Shands Hospital, University of Florida, from June 1991 to July 1992. This facility serves a largely rural, indigent patient population. The 390 patients enrolled in the study had previously uncomplicated pregnancies and were in labor when admitted to the labor and delivery unit. Patients were excluded if they had received antibiotic treatment 2 weeks prior to enrollment. Written informed consent was obtained from each patient in accordance with guidelines established by the Institutional Review Board.

Vaginal secretions were collected from each patient by rolling a rayon-tipped swab (Culturette Collection and Transport System, Becton-Dickinson, Cockeysville, MD) along the vaginal wall. The swab was smeared directly onto a clean glass slide. The slides were air-dried, heat-fixed, and stained in batches. The standard procedure for the Gram's stain was followed. The stained slides were examined microscopically under an oil-immersion objective and interpreted according to the standardized scoring system developed by Nugent et al.[7] Briefly, each slide was examined for the following morphotypes: large gram-positive rods (*Lactobacillus* morphotypes), small gram-variable and gram-negative rods (*Gardnerella* and *Bacteroids* morphotypes), and curved gram-variable rods (*Mobiluncus* morphotypes). Each morphotype was quantitated as 0 to 4+ and weighted to yield a score of 0 to 10.[7] A vaginal smear with a score of ≥7 was defined as BV, a score of 4–6 was classified as intermediate BV, and a score of 0–3 was considered normal.

Three hundred forty of these women were also screened for carriage of group B streptococci by placing a rayon-tipped applicator in the lower vagina and swabbing it along the perineum and the perirectal area. Specimens were subsequently cultured in selected Todd-Hewitt broth as previously described.[8]

When all of the Gram's stains had been classified, the hospital charts of the 390 mothers were reviewed to collect pertinent obstetric data and to determine the frequency of chorioamnionitis and endometritis. The diagnoses of intrapartum and postpartum infection were established on the basis of commonly accepted clinical criteria such as maternal fever, maternal tachycardia, fetal tachycardia, uterine tenderness, discolored amniotic fluid or purulent lochia, and no other localizing sign of infection. For the assessment of neonatal outcome, newborn charts were reviewed for the diagnosis of suspected or culture-proven sepsis (positive blood, urine, or cerebrospinal fluid culture). The association between BV and each maternal factor was analyzed using the chi-square test for categorical variables. Odds ratios (ORs) and their 95% confidence intervals (CIs) are also reported for these comparisons. For comparisons of the difference between mean values, the two-tailed, unpaired t test was utilized. $P \leq 0.05$ was considered significant.

### RESULTS

Maternal demographic and obstetric characteristics are presented in Table 1. The prevalence of BV in this population was 116/390 (30%, 95% CI, 25.5–34.5%). Sixteen percent of the patients had intermediate Gram's stains, and 54% had normal Gram's stains. Eighty-eight percent of women were term, and 12% were preterm. The frequency of BV was similar in these patients. Variables such as parity, status of the membranes, duration of labor, number of vaginal examinations, duration of ruptured membranes, length of fetal monitoring, and mode of delivery were similar in the 3 groups. BV was significantly more prevalent in nonwhites (37%) than whites (25%, $P = 0.005$).

Forty-seven of 390 (12%) patients developed either chorioamnionitis or endometritis. Nineteen of the 116 (16.4%) women with BV developed peripartum infection compared with 17/211 women with normal Gram's stains [8.1%, $P = 0.034$, OR = 2.0 (95% CI, 1.07–3.73)]. In patients with intermediate Gram's stains, the frequency of infection was also increased (11/63, 17.5%) compared with normal women [$P = 0.054$, or = 2.1 (95% CI, 1.12–3.94)]. The sensitivity of BV as a single

**TABLE 1. Demographic and clinical characteristics of study patients**

| Variable | Normal (N = 211) | BV (N = 116) | Intermediate Gram's stain (N = 63) | *P* value |
|---|---|---|---|---|
| Mean age (y) | 23.8 ± 6 | 22.7 ± 6 | 24.1 ± 7 | NS |
| Gestational age | | | | NS |
| > 36 weeks | 186 | 100 | 57 | |
| ≤ 36 weeks | 25 | 16 | 6 | |
| Race | | | | 0.005* |
| White | 141 | 59 | 37 | |
| Non-White | 70 | 57 | 26 | |
| Parity | | | | NS |
| Nulliparous | 86 | 45 | 21 | |
| Multiparous | 125 | 71 | 42 | |
| Membranes | | | | NS |
| Intact | 135 | 71 | 35 | |
| Ruptured | 76 | 45 | 28 | |
| Duration of membrane rupture (h) | | | | NS |
| 0–8 | 130 | 66 | 33 | |
| 9–16 | 51 | 37 | 18 | |
| > 16 | 31 | 13 | 12 | |
| Number of vaginal examinations | | | | NS |
| ≤ 6 | 129 | 72 | 40 | |
| > 6 | 82 | 44 | 22 | |
| Duration of labor (h)[a] | | | | NS |
| 0–8 | 51 | 30 | 16 | |
| 9–16 | 88 | 41 | 24 | |
| > 16 | 37 | 24 | 15 | |
| Duration of fetal monitoring (h)[a] | | | | NS |
| 0–8 | 174 | 90 | 44 | |
| 9–16 | 30 | 21 | 12 | |
| Mode of delivery | | | | NS |
| Vaginal | 178 | 97 | 56 | |
| Cesarean | 33 | 19 | 7 | |
| Group B streptococcal colonization (colonized/screened) | 28/180 | 21/105 | 16/55 | 0.039** |

[a]Includes only those patients with complete data in their hospital charts.
*BV vs. normal flora.
**Intermediate vs. normal flora.

risk factor for peripartum infection was 40% (19/47), and the specificity was 71% (246/343). The positive and negative predictive values were 16% (19/116) and 92% (246/274), respectively.

Four women with chorioamnionitis and endometritis were bacteremic. Three of these women had BV. In 2 of the 3, the bacteremias were caused by multiple anaerobic organisms. Sixty-one of 392 (15.6%) neonates developed suspected (55) or proven (6) sepsis. Twenty-two (36.1%), 15 (24.6%), and 24 (39.3%), respectively, were delivered to mothers with BV, intermediate Gram's stains, and normal vaginal flora. There was no significant difference in the incidence of suspected or proven neonatal infection in infants born to mothers with BV (22/116, 18.9%) compared with those born to mothers with normal Gram's stains (24/213, 12.7%). However, infants delivered to mothers with intermediate Gram's stains had a significantly higher incidence of suspected or proven infection (15/63, 24%) compared with those delivered to women with normal Gram's stains [$P = 0.021$, OR = 2.2 (95% CI, 1.26–3.76)]. This finding most likely occurred because women with intermediate Gram's stains were more likely than women with normal Gram's stains to be colonized with group B streptococci [29% vs. 16%, $P = 0.039$, OR = 1.8 (95% CI, 1.06–3.05)]. Of the proven cases of neonatal sepsis, 4 of the 6 infants had blood cultures positive for group B streptococci. One had a culture positive for both group B streptococci and *Klebsiella pneumoniae*, and one had a culture positive for *Escherichia coli*.

### DISCUSSION

The diagnosis of BV is typically based on the presence of a homogeneous, malodorous, gray vaginal discharge with a pH >4.5, a positive amine test, and clue cells on microscopy. These clinical criteria have been reported to correlate well with the results of a Gram's stain of vaginal fluids that is interpreted according to the criteria of Spiegel et al.[9] A high level of reproducibility and reliability in identifying BV has resulted when Spiegel's criteria have been used in conjunction with the standardized scoring system proposed by Nugent et al.[6,7,10,11] Since the change from a normal microbial profile to that characteristic of BV can be reliably evaluated by Gram's stain, the diagnosis of BV in our study was based solely on this method.

In this population of women whose pregnancies were uncomplicated, at least until the time of admission, we found that those with BV were at higher risk of developing chorioamnionitis and endometritis. This association remained evident after considering other recognized risk factors for infection such as duration of labor, duration of ruptured membranes, length of fetal monitoring, number of vaginal examinations, gestational age, and mode of delivery. The relatively high bacterial concentrations and the shift to the more virulent facultative and anaerobic microorganisms in the vaginas of patients with BV appear to increase the possibility of ascending infection.[12] Previous investigations have shown that bacterial enzymes such as proteases, lipases, and sialidases are present in high concentrations in the vaginal fluids of women with BV. These enzymes may facilitate the attachment of organisms to mucosal cells of the genital tract and subsequent invasion into the soft tissue of the pelvis.[13]

Although BV was associated with an increased risk of maternal infection, BV flora was not isolated from the neonates with proven infection. The increased incidence of neonatal sepsis in mothers with intermediate Gram's stains correlated with the high rate of maternal group B streptococcal colonization in these women. The primary isolate in 5/6 cases of proven sepsis was group B streptococci, an organism that is not directly associated with the pathogenesis of BV. The other 2 blood culture isolates were *E. coli* and *K. pneumonia*, neither of which is a major pathogen in patients with BV.

Our findings raise several major questions that merit further investigation. In view of the association between BV and peripartum infection, should pregnant women be routinely screened for BV? If so, what is the optimal time for screening? Should infected women be treated prior to delivery? Finally, should prophylatic antibiotics administered at the time of cesarean delivery specifically target the polymicrobial flora of BV?

### REFERENCES

1. Spiegel CA, Amsel R, Eschenbach D, Schoenknecht F, Holmes KK: Anaerobic bacteria in nonspecific vaginitis. N Engl J Med 303:601–607, 1980.
2. Spiegel CA: Bacterial vaginosis. Clin Microbiol Rev 4:485–502, 1991.
3. Krohn MA, Hillier SL, Lee ML, Eschenbach DA: Vaginal *Bacteroides* species are associated with an increased rate of preterm delivery among women in preterm labor. J Infect Dis 164:88–93, 1991.
4. Martius J, Krohn MA, Hillier SL, Stamm WE, Holmes KK, Eschenbach DA: Relationship of vaginal *Lactobacillus* species, cervical *Chlamydia trachomatis*, and bacterial vaginosis to preterm delivery. Obstet Gynecol 71:89–95, 1988.
5. Gravett MG, Hummel D, Eschenbach DA, Holmnes KK: Preterm labor associated with subclinical amniotic fluid infection and with bacterial vaginosis. Obstet Gynecol 67:229–237, 1986.
6. Watts HD, Krohn MA, Hillier SL, Eschenbach DA: Bacterial vaginosis as a risk factor for postcesarean endometritis. Obstet Gynecol 75:52–58, 1990.
7. Nugent RP, Krohn MA, Hillier SL: Reliability of diagnosing bacterial vaginosis is improved by a standardized method of gram stain interpretation. J Clin Microbiol 29:297–301, 1991.
8. Clark P, Armer T, Duff P, Davidson K: Assessment of a rapid latex agglutination test for group B streptococcal colonization of the genital tract. Obstet Gynecol 79:358–363, 1992.
9. Spiegel CA, Amsel R, Holmes KK: Diagnosis of bacterial vaginosis by direct gram stain of vaginal fluid. J Clin Microbiol 18:170–177, 1983.
10. Mazzuli T, Andrew ES, Donald EL: Reproducibility of interpretation of gram-stained vaginal smears for the diagnosis of bacterial vaginosis. J Clin Microbiol 28:1506–1508, 1990.
11. Jeosoff MR, Hillier SL, Josodiwondo S, Linnan M: Reproducibility of a scoring system for gram stain diagnosis of bacterial vaginosis. J Clin Microbiol 29:1730–1731, 1991.

12. Silver HM, Sperling RS, St. Clair P, Gibbs R: Evidence relating bacterial vaginosis to intramniotic infection. Am J Obstet Gynecol 161:808–812, 1989.
13. Briselden AM, Moncla BJ, Stevens CE, Hillier SL: Sialides (neuraminidases) in bacterial vaginosis and bacterial vaginosis-associated microflora. J Clin Microbiol 30: 663–666, 1992.
